# A multifunctional octacalcium phosphate pentahydrate with dual environmental and biomedical functions: efficient dye removal, potent antimicrobial activity, and ionic regulation in physiological media

**DOI:** 10.1039/d6ra01926a

**Published:** 2026-07-02

**Authors:** Mohammed Zerrouk, Imane Haydari, Mohammed Mesrar, Belkheir Hammouti, Mohammed Lachkar, Lamy Mamdoh Mohamed Hamed, Ahmad El-Harairy, Khalil Azzaoui, Rachid Ouarsal

**Affiliations:** a Engineering Laboratory of Organometallic, Molecular Materials, and Environment, Faculty of Sciences, University Sidi Mohamed Ben Abdellah Fez 30000 Morocco Mohammed.zerrouk@usmba.ac.ma; b Materials Science, Energy and Nanoengineering (MSN) Department, Mohammed VI Polytechnic University Ben Guerir Morocco; c Signals, Systems and Components Laboratory (LSSC), Faculty of Sciences and Technologies of Fez, Sidi Mohamed Ben Abdellah University B. P. 2022 Fez Morocco; d Euro-Mediterranean University of Fes BP 15 Fes 30070 Morocco; e Department of Environment and Agricultural Natural Resources, College of Agricultural and Food Sciences, King Faisal University Al-Ahsa Saudi Arabia lamy.hamed@kfu.edu.sa; f Department of Chemical and Biomolecular Engineering, College of Engineering, University of Nebraska-Lincoln Lincoln NE 68588 USA ael-harairy2@nebraska.edu; g Department of Soils and Water, Faculty of Agriculture, Damietta University New Damietta Damietta 34517 Egypt; h Laboratory of Industrial Engineering, Energy and the Environment (LI3E), SUPMTI Rabat Morocco

## Abstract

Ca_8_(HPO_4_)_2_(PO_4_)_4_·5H_2_O, (OCP), has been successfully synthesized *via* a wet chemical precipitation method. The analysis of the structure and identification of the composition were performed using X-ray diffraction (XRD), and Rietveld refinement was applied to confirm phase purity. The refined results indicated the formation of highly crystalline octacalcium phosphate with no detectable secondary phases. Fourier-transform infrared spectroscopy (FTIR) confirmed the presence of characteristic phosphate functional groups and water molecules. TGA shows that the thermal decomposition of the synthesized OCP leads to the release of water molecules, while scanning electron microscopy (SEM) revealed a plate-like morphology and a homogeneous calcium-to-phosphorus distribution, supporting its suitability for antimicrobial applications. Hirshfeld surface analysis (HSA) indicates that the structural integrity of OCP pentahydrate is predominantly governed by extensive hydrogen bonding. The synthesized material showed a BET surface area of 87.80 m^2^ g^−1^ and favorable adsorption for methylene blue dye (MB) from aqueous solutions, with an adsorption capacity of 8.05 mg g^−1^ for an initial concentration of 10 ppm. Thermodynamic studies show that the sorption of MB onto OCP is spontaneous and endothermic. The sorption kinetics of MB on OCP fit the pseudo-second-order model better than the pseudo-first-order model, resulting in a higher correlation coefficient (*R*^2^ > 0.99). OCP demonstrated potent bactericidal activity against all tested bacteria, with MIC and MBC values ranging from 0.14–0.28 mg mL^−1^ and 0.29–0.56 mg mL^−1^, respectively, exhibiting the highest efficacy against *S. aureus*, and *B. cereus*. Furthermore, the ionic concentrations in simulated body fluid (SBF) were monitored in the presence of the synthesized material to evaluate its bioactivity.

## Abstract 2

Ca_8_(HPO_4_)_2_(PO_4_)_4_·5H_2_O, (OCP), has been successfully synthesized *via* a wet chemical precipitation method.

## Graphical abstract

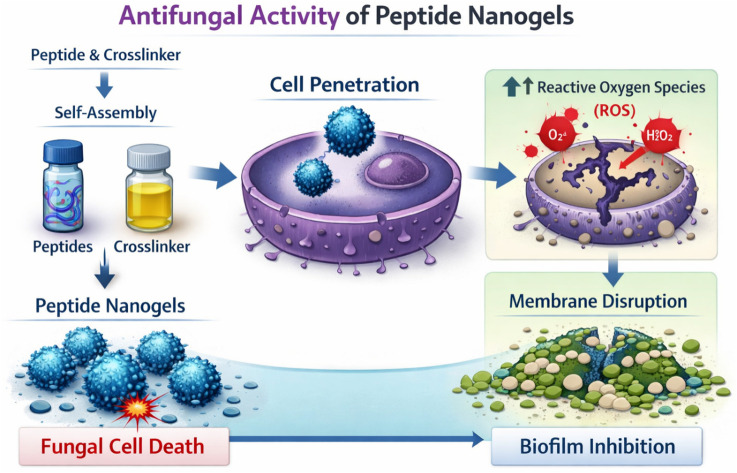

## 1 Introduction

Calcium phosphate compounds have garnered considerable interest due to their chemical stability, low toxicity, and high binding affinity to metal surfaces. Among them, octacalcium phosphate (OCP), commonly recognized as a precursor of hydroxyapatite, exhibits unique structural and physicochemical properties, including a layered crystal structure, high surface activity, and the presence of reactive phosphate groups. The synthesis of octacalcium phosphate (OCP) is of considerable scholarly interest owing to its multifaceted functional properties and extensive range of applications.^1,2^ Initially, OCP has garnered attention for its capacity to adsorb dyes and various pollutants, as its surface characteristics and layered architecture facilitate interactions with a multitude of organic molecules, placing it as a potential candidate for the treatment of contaminated water and water pollution control. Paints and dyes are used extensively in the textile, leather, paper, and other industries, and their environmental release can have harmful effects on both aquatic life and human health. Phosphate materials have a high surface area and porosity, allowing for efficient dye adsorption.^3^ Additionally, the presence of phosphate functional groups on the surface of these materials enhances their affinity for the dyes.^4,5^

Moreover, OCP bears structural resemblance to biological apatite and functions as a precursor to hydroxyapatite, which constitutes the main mineral component of bones and dentes.^6–8^ The controlled synthesis of OCP permits meticulous regulation of particle size, morphology, and crystallinity, factors that are essential for the optimization of its performance. OCP is subject to extensive investigation in the realms of bone tissue engineering, implant coatings, and drug delivery systems, attributable to its remarkable biocompatibility, bioactivity, and gradual conversion into hydroxyapatite under physiological conditions.^9,10^ Its capacity to incorporate therapeutic ions or bioactive molecules further enhances its applicability in regenerative medicine and controlled release systems, underscoring the critical need for the development of reproducible and customized synthesis strategies.

In addition, calcium phosphate-based materials are progressively transitioning from being merely passive, osteoconductive scaffolds to becoming dynamic platforms designed for proactive infection management.^11^ The deliberate synthesis of these compounds, whether aimed at producing stoichiometric hydroxyapatite or calcium-deficient, amorphous phases, fundamentally influences their chemical reactivity and biological interactions. This intrinsic adaptability is pivotal to their nascent function in antimicrobial strategies, effectively addressing the pressing issue of implant-associated and drug-resistant infections. By acting as a reservoir for therapeutic ions or as a degradable substrate facilitating localized drug delivery, engineered calcium phosphates present a targeted and multifaceted strategy for pathogen suppression.^12^

Their efficacy in antibacterial applications is extensively substantiated, particularly when modified with ionic dopants.^13^ These enhanced ceramics function through a robust amalgamation of mechanisms: the prolonged “ion-burst” release that interferes with cellular processes, the catalytic production of cytotoxic reactive oxygen species, and direct nano-scale interactions that undermine microbial structural integrity.^14^ Concurrently, the investigation of calcium phosphate in antifungal applications offers a compelling, albeit less explored, avenue. Fungal cells, characterized by their unique chitin-based cell walls, are vulnerable to analogous physicochemical assaults. Calcium phosphate coatings can significantly inhibit fungal adhesion and biofilm development on biomedical surfaces, while their composite forms augment the stability and localized release of traditional antifungal agents, thereby minimizing systemic toxicity.^15^

The multifunctional attributes of octacalcium phosphate (OCP) are predominantly dictated by its unique layered crystalline architecture and elevated surface reactivity,^16^ which together augment its adsorption, antimicrobial, and bioactive properties. OCP is characterized by alternating layers resembling apatite and hydrated layers, which yield a substantial specific surface area and a plethora of active sites, enabling robust interactions with organic dye molecules *via* electrostatic forces and surface complexation, thereby elucidating its notable adsorption efficacy.^17^ Moreover, the ion-exchange capacity of OCP facilitates the release and absorption of Ca^2+^ and PO_4_^3−^ ions, thereby contributing to antimicrobial mechanisms by modifying the local ionic milieu and impeding microbial growth. This ion exchange mechanism is also pivotal in biomedical contexts, as the liberated ions modulate mineralization processes and foster apatite synthesis in simulated body fluid (SBF), consequently enhancing bioactivity and osteoconductivity. Thus, the interplay between OCP's structural attributes, surface chemistry, and ion-regulatory capacity constitutes the fundamental rationale for its adaptability in both environmental and biomedical domains.^18^

The increasing discharge of industrial effluents containing dyes, heavy metals, pharmaceuticals, and other hazardous contaminants has become a major environmental concern worldwide.^19^ Various wastewater treatment technologies have been developed to address this issue, including coagulation–flocculation, chemical precipitation, membrane filtration, advanced oxidation processes, catalytic degradation,^20^ biological treatment, ion exchange, and adsorption.^21,22^ Although many of these methods are effective, they often suffer from limitations such as high operational costs, sludge generation, incomplete pollutant removal, or complex operating conditions. Among them, adsorption has emerged as one of the most attractive treatment approaches owing to its simplicity, high efficiency, low energy requirements, and applicability to a wide range of pollutants. Consequently, considerable efforts have been devoted to developing novel adsorbent materials with enhanced adsorption capacity, environmental compatibility, and cost-effectiveness.

The present work reports the synthesis of phase-pure OCP through a simple wet-precipitation route and provides a comprehensive evaluation of its intrinsic multifunctional properties without any chemical modification. The novelty of this study does not reside in the synthesis method itself, but rather in the integrated assessment of the adsorption of methylene blue, antimicrobial activity, and *in vitro* bioactivity using a single phase-pure OCP material. By establishing the fundamental performance of pristine OCP across environmental and biomedical applications. This work provides valuable baseline data for future comparisons with engineered OCP-based materials and contributes to a better understanding of the intrinsic capabilities of this calcium phosphate phase. In this context, X-ray diffraction combined with Rietveld refinement provides a powerful tool for accurate phase identification, crystallographic parameter determination, and verification of material purity. Complementary techniques such as FTIR spectroscopy, electronic scanning microscopy (SEM), and Brunauer–Emmett–Teller analysis (BET) provide further insight into functional groups, morphology, Surface area, and porosity of the synthesized material.

## 2 Experimental

### 2.1 Products

The products and solvents were bought from Sigma-Aldrich and used as provided, without any additional purification.

### 2.2 Synthesis

The porous Ca_8_(HPO_4_)_2_(PO_4_)_4_·5H_2_O, was obtained utilizing a wet chemical methodology in accordance with a previously documented protocol. In summary, two distinct aqueous solutions were prepared. Solution 1 comprised 14.8 g of Ca(NO_3_)_2_·4H_2_O dissolved in 0.2 L of deionized water, whereas Solution 2 comprised 13.8 g of (NH_4_)_2_HPO_4_ dissolved in 0.1 L of deionized water. Both solutions underwent stirring for 90 minutes at ambient temperature until the complete dissolution of the chemical constituents was achieved. The pH of the reaction mixture was subsequently and promptly adjusted to 7 through the incorporation of ammonium hydroxide (NH_4_OH), resulting in the emergence of a white precipitate. The resultant precipitate was subjected to filtration, subsequently washed several times with deionized water, then dried in an oven at a temperature of 60 °C for a period of 24 hours.

### 2.3 X-ray analysis

The phase composition of the deposited powder, OCP, was meticulously examined utilizing a Shimadzu XRD-6000 diffractometer, which operates with CuKα1 radiation (*λ* = 1.5406 Å). By employing a precise step of 0.02° and a scan range of 2*θ* ∼ 10°–80°, the instrument furnished comprehensive data that is indispensable for our thorough analysis of phase compositions. The evaluation of phase purity was conducted through Rietveld refinement.

### 2.4 FTIR spectroscopy

The infrared spectrum was acquired using a Shimadzu FTIR-8400S spectrometer to discern the structural groups in the Ca_8_(HPO_4_)_2_(PO_4_)_4_·5H_2_O. This analysis was performed utilizing the KBr pellet method, achieving a resolution of 2 cm^−1^ across the wavelength range of 4000–450 cm^−1^.

### 2.5 Scanning electron microscopy

The surface morphology of the obtained powder was examined using a Quanta 200 FEG SEM microscope. This provided detailed images of the composite's surface structure, which are crucial for understanding the material's texture.

### 2.6 Brunauer–Emmett–Teller analysis

The measurements of porosity and surface area were carried out using a TriStar II Plus Micromeritics analyzer (Version 3.03; Micromeritics Instrument Corp., USA). Data were collected using Unit 1, Port 3 (Serial no. 944), and analyzed with the manufacturer's software.

### 2.7 Hirshfeld surface analysis

Hirshfeld surfaces, which were mapped using *d*_norm_ alongside two-dimensional representations of OCP fingerprints, were generated using CrystalExplorer,^23^ facilitating the identification of regions particularly important for intermolecular interactions; the scaled contact distance (*d*_norm_) is a function of both *d*_e_ and *d*_i_ (where *d*_e_ is the distance from the surface to its closest atom on its outside, whereas *d*_i_ refers to the distance from the surface to its closest atom on its inside), as well as *r*^vdW^, which are van der Waals radii, as shown in eqn (1):1
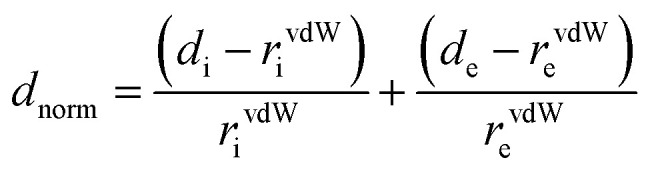


Hirshfeld's molecular surface plots, colored with *d*_norm_, utilize a red-white-blue color scheme where red corresponds to the closest contacts between two molecules, white indicates the distances corresponding to the separation at van der Waals radii, and blue identifies longer contacts – all of this in an effort to highlight regions particularly relevant for intermolecular interactions. Furthermore, we include other graphical representations showing the shape index and curvature from local curvatures.

### 2.8 Removal of MB dye

The discharge of colored effluents into the environment not only leads to environmental pollution but also interferes with numerous biological cycles.^4^ Because of their stability and poor biodegradability, these emissions pose a serious risk to both man and the environment.^24,25^ The adsorption performance of the synthesized OCP toward MB was investigated through batch adsorption experiments. A stock solution of methylene blue was prepared using distilled water and subsequently diluted to the desired concentrations. The effect of solution pH on MB adsorption was evaluated over a pH range of 2–12. The pH was adjusted using diluted HCl or NaOH solutions. The adsorption kinetics were studied by contacting the adsorbent with the MB solution for 180 min. Aliquots were withdrawn every 20 min, centrifuged to separate the adsorbent particles, and analyzed for the residual dye concentration using a UV-Vis spectrophotometer at the maximum absorption wavelength of MB. The effect of the initial dye concentration was investigated using methylene blue solutions with concentrations ranging from 2 to 10 mg L^−1^ under optimized adsorption conditions. To evaluate the influence of temperature, adsorption experiments were performed at 25, 40, 50, and 60 °C.

**Fig. 1:**
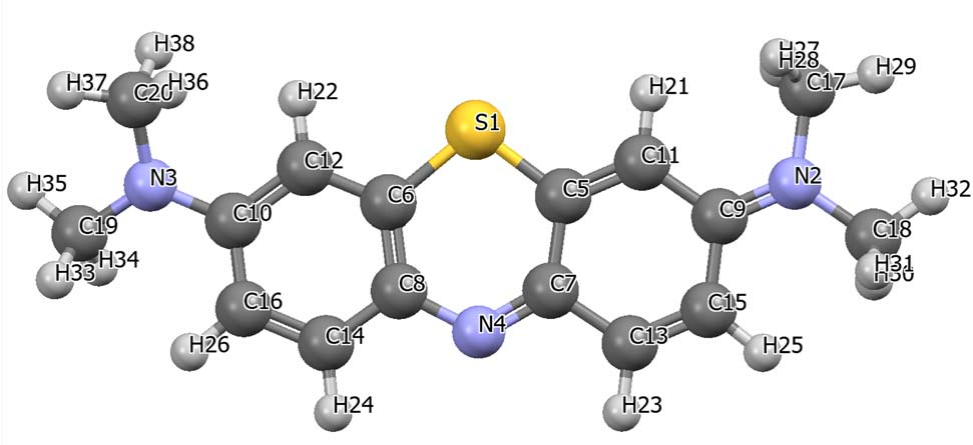
Methylene blue (MB).

The Ca_8_(HPO_4_)_2_(PO_4_)_4_·5H_2_O adsorption capability towards dye was also quantified using the Langmuir and the Freundlich isotherms (Table 1). Methylene blue (MB) (a cationic dye) (Fig. 1) is used as the model pollutant. The dyes are dissolved in distilled water to the desired concentrations.

**Table 1:** Isothermal models

Model	Equation	No.
Langmuir	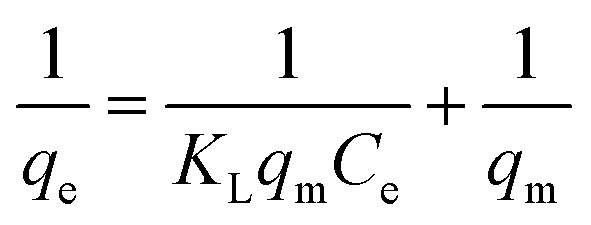	(4)
Freundlich	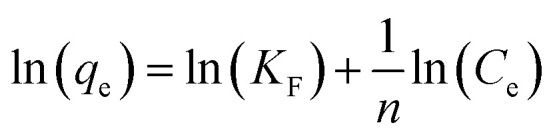	(5)

Absorbance of treated solutions was evaluated using a JASCO V-630 spectrophotometer, a UV-Visible type of spectrometer. Absorption maximum of MB is 665 nm. The *q*_e_ (mg g^−1^) and the adsorption percentage *R* (%) were determined based on eqn (2) and (3):2
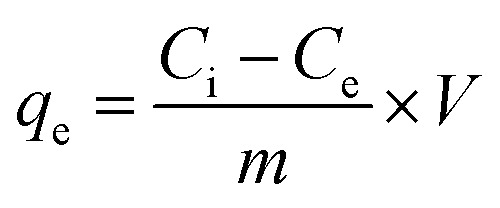
3
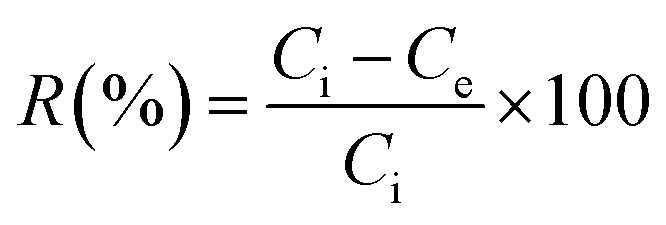


### 2.9 Assessment of antibacterial activity

#### 2.9.1 Bacterial cultures

The antibacterial activity was assessed on a variety of Gram (+) and Gram (−) bacteria that were selected based on their clinical significance and pathogenicity potential. The strains utilized in the current study are summarized in Table 2.

**Table 2:** Bacterial strains

Species	Gram reaction
*E. cloacae* (clinical isolate)	Gram (−)
*S. typhimurium* (ATCC 13311)	
*E. coli* (ATCC 35210)	
*P. aeruginosa* (ATCC 27853)	
*M. flavus* (ATCC 10240)	Gram (+)
*L. monocytogenes* (NCTC 7973)	
*S. aureus* (ATCC 6538)	
*B. cereus* (clinical isolate)	

#### 2.9.2 Determination of MIC and MBC

The broth microdilution method in 96-well microplates^26,27^ was used to determine antibacterial activity. Bacterial suspensions were constructed in sterile saline and standardized to a final concentration of 10 000 CFU mL^−1^, while the tested compound was solubilized into 5% dimethyl sulfoxide (DMSO), forming a stock solution at a concentration of 1 mg mL^−1^ containing Tween80 to enhance solubility. Aliquots of tryptic soy broth (TSB, 100 µL) were added to each well, and the bacterial inoculum was added to reach a final density of 10 000 CFU per well.^27^ The plates were incubated for the appropriate time.

The MIC was defined as the lowest concentration remaining without visible growth of bacteria. The viability of the bacteria was additionally assessed using a colorimetric assay based on iodonitrotetrazolium chloride (INT) reduction. Minimum Bactericidal Concentration (MBC) was determined by transferring 2 µL from growth-free wells into fresh TSB (100 µL per well) and incubating for 24 h. The minimum bactericidal concentration (MBC) was defined as the lowest concentration that gave no detectable growth of bacteria, which corresponded to a 99.5% reduction of the initial inoculum. The optical density was read at 655 nm through a Microplate Manager 4.0 system (Bio-Rad Laboratories, Hercules, CA, USA). Positive controls used were ampicillin (69–1100 µmol mL^−1^) and streptomycin (687–2290 µmol mL^−1^), while 5% aqueous DMSO served as the negative control. All antibacterial experiments were performed in triplicate under identical conditions. MIC and MBC values are reported as mean ± standard deviation (SD) of three independent determinations.

### 2.10 Antifungal test

The antifungal efficacy of octacalcium phosphate (OCP) was systematically assessed utilizing the broth microdilution technique against various filamentous fungi, specifically *Aspergillus fumigatus*, *A. versicolor*, *A. niger*, *Penicillium ochrochloron*, *P. funiculosum*, *P. verrucosum*, and *Trichoderma viride*. RPMI-1640 medium was utilized as the culture medium, and OCP was prepared through serial two-fold dilutions (ranging from 0.78 to 0.18 mg mL^−1^ and lower) within 96-well microplates. Dimethyl sulfoxide (DMSO) was employed as a solvent, maintaining a final concentration not exceeding 5%. The fungal inocula were standardized and subsequently introduced into each well, with the exception of control wells. Growth controls, comprising medium supplemented with inoculum, and sterility controls, consisting solely of medium, were incorporated into the experimental design, while bifonazole and ketoconazole were utilized as reference antifungal agents. The plates were subjected to incubation at 35 °C for a duration of 48 hours, during which antifungal activity was evaluated through both visual inspection and spectrophotometric analysis. The minimum inhibitory concentration (MIC) was defined as the lowest concentration of OCP that effectively inhibits visible fungal growth, while the minimum fungicidal concentration (MFC) was ascertained by subculturing from wells exhibiting no growth onto agar plates. All antifungal assays were conducted in triplicate under identical experimental conditions. The MIC and MFC values are expressed as mean ± standard deviation (SD) of three independent determinations, and the reported error margins correspond to the calculated standard deviations.

### 2.11 Investigation of how synthetic biomaterials release active chemicals

The ability to interchange ions with the surrounding immersive environment is essential for evaluating a biocompatible material's potential to promote tissue adhesion. Consequently, *in vitro* assessments are conducted to examine this capability. These evaluations involve the immersion of the sample in a synthetic physiological solution known as SBF (Simulated Body Fluid), the ionic composition of which is meticulously calibrated to approximate that of human blood plasma.^15^ The SBF is set to maintain a pH of 7.4. Table 3 delineates a comparative analysis of the ionic SBF concentrations in relation to those in human blood plasma. The multifunctional biomaterials under investigation were predominantly synthesized in the form of fine powder. This specific morphological configuration was selected to facilitate an accurate assessment of the kinetics of bioactivity of the biomaterial synthesized at various temporal intervals. For the *in vitro* experiments, OCP sample was put in a vial that was hermetically sealed and submerged in 60 mL of simulated body fluid (SBF). In the incubator, the temperature was carefully maintained at 37 °C with regulated agitation. For the powdered sample, the immersion times were set at 5, 7, 14, 21, and 37 days. The ionic concentrations were analyzed using ICP-AES.

**Table 3:** Ionic concentrations of SBF and human blood plasma in mM (ref. 15)

Ions (mM)	Na^+^	Cl^−^	HCO^3−^	HPO_4_^2−^	K^+^	Mg^2+^	Ca^2+^	SO_4_^2−^
SBF	142.0	147.8	4.2	1.0	5.0	1.5	2.5	0.5
Plasma	142.0	103.0	27.0	1.0	5.0	1.5	2.5	0.5

## 3 Results

### 3.1 XRPD and Rietveld refinement analysis

**Fig. 2:**
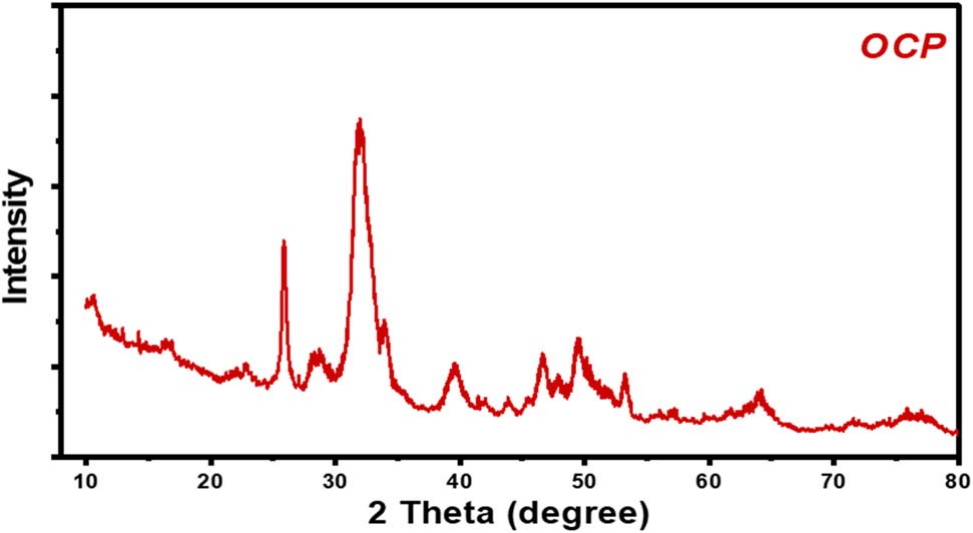
XRPD pattern of the as-synthesized OCP powder before Rietveld refinement.

**Fig. 3:**
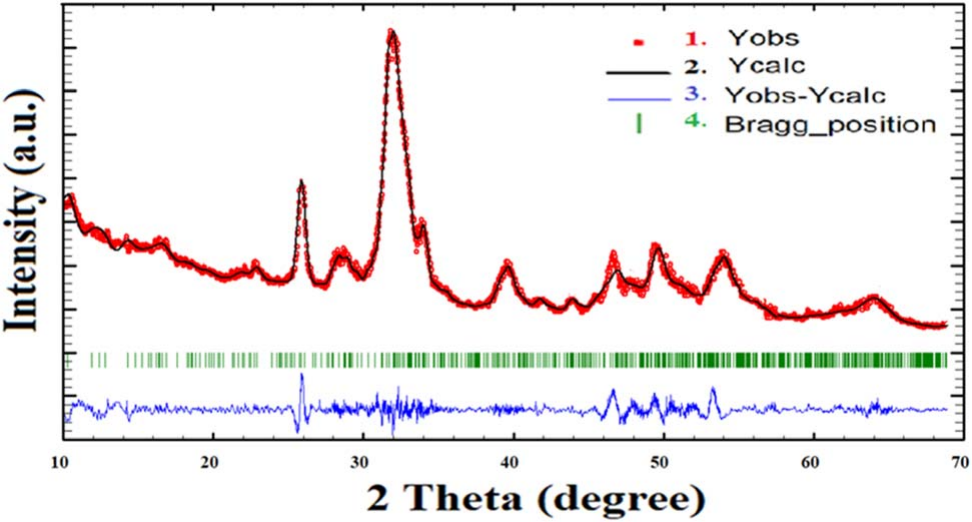
Final Rietveld refinement plot of OCP. The observed (red circles), calculated (black line), and difference (blue line) profiles are shown. Vertical green ticks indicate the position of Bragg reflections.

The phase composition of the synthesized powder was scrutinized through X-ray powder diffraction (XRPD) employing CuKα radiation (*λ* = 1.5406 Å) within the 2*θ* range of 10°–80° (Fig. 2). The resultant diffraction pattern displays characteristic reflections of octacalcium phosphate (OCP), thereby substantiating the successful synthesis of this phase. A low-angle reflection noted at 2*θ* ≈ 10.6° serves as a distinctive hallmark of OCP and is correlated with its layered crystalline architecture, indicating the existence of hydrated interlayer planes. The most pronounced diffraction peaks manifest at 2*θ* ≈ 31.67° (*d* ≈ 2.83 Å) and 32.19° (*d* ≈ 2.78 Å), which correspond to the principal crystallographic planes of OCP. Supplementary reflections positioned at 25.84°, 28.12°, 28.79°, 34.02°, 39.81°, 46.63°, and 49.55° further corroborate the identification of the OCP phase, showing strong concordance with standard reference data from the JCPDS database (PDF no. 26-1056). No discernible diffraction peaks corresponding to secondary calcium phosphate phases, such as stoichiometric hydroxyapatite or tricalcium phosphate, were observed, thereby suggesting that the produced substance has a high level of phase purity. The moderate broadening of the diffraction peaks implies a medium level of crystallinity, which aligns with the metastable characteristics of octacalcium phosphate (OCP) and with materials synthesized *via* wet chemical methodologies. To validate the phase identification and to refine the structural parameters, a Rietveld refinement analysis was executed utilizing the FullProf software, presuming an orthorhombic lattice structure with the space group *Pmmm*, as documented in the JCPDS reference for OCP. The refinement yielded a satisfactory quality of fit, with *R*_p_ = 4.04%, *R*_wp_ = 5.74%, *R*_exp_ = 5.38%, and *χ*^2^ = 1.14, based on 622 reflections acquired in the 2*θ* range of 10.13°–79.99°, with a step size of 0.017° employing CuKα radiation (*λ* = 1.5406 Å) (Fig. 3).

The refined unit cell parameters are determined to be *a* = 22.3237 Å, *b* = 18.5968 Å, *c* = 5.6379 Å, which yield a unit cell volume of *V* = 2340.58 Å^3^, aligning closely with the values documented in the JCPDS card and existing literature pertaining to octacalcium phosphate. The symmetry operations associated with the *Pmmm* space group effectively replicate the equivalent atomic positions. The Rietveld refinement substantiates the existence of a weakly crystallized, anisotropic, and hydrated OCP phase, which maintains its lamellar architecture, devoid of any discernible secondary phases. These findings reinforce the qualitative XRD analysis, peak indexing, and juxtaposition with JCPDS reference data.

### 3.2 FTIR spectroscopy

**Fig. 4:**
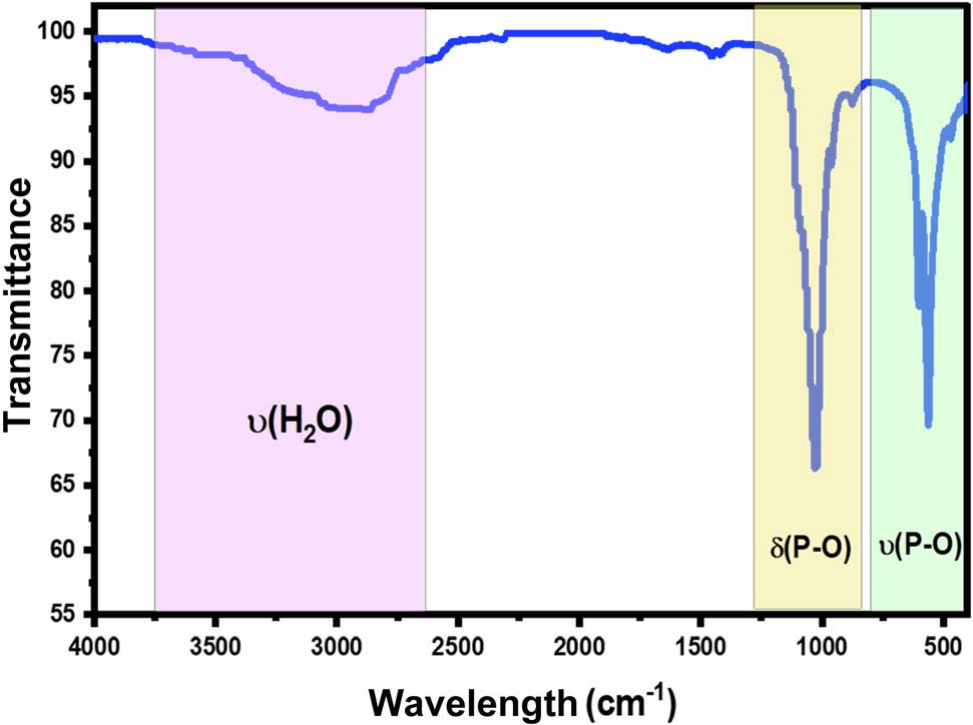
Infrared spectrum of the synthesized product.

The FT-IR spectrum of octacalcium phosphate (OCP) (Fig. 4) shows the characteristic vibrational features of structural water, orthophosphate (PO_4_^3−^) and hydrogen phosphate (HPO_4_^2−^) groups, confirming the hydrated phase. A broad and intense absorption band in the 3600–2800 cm^−1^ region is attributed to O–H stretching vibrations of crystallization water and hydrogen-bonded hydroxyl groups.^27^ Strong absorption bands between 1150 and 900 cm^−1^ arise from P–O stretching vibrations of both PO_4_^3−^ and HPO_4_^2−^ units;^28–30^ the multiplicity and broadening of these bands reflect the coexistence of the two phosphate species within the OCP lattice.^4^ A distinct band around 875 cm^−1^ is assigned to the P–OH vibration of the hydrogen phosphate group, which is a key spectral signature distinguishing OCP from hydroxyapatite. Bands observed in the 600–500 cm^−1^ region correspond to O–P–O bending modes typical of crystalline calcium phosphates, and the band near 470 cm^−1^ is attributed to the *ν*_2_ bending vibration of PO_4_^3−^ tetrahedra. Overall, the combination of these characteristic absorptions confirms the formation of well-structured, hydrated octacalcium phosphate.

### 3.3 TGA-DTA

**Fig. 5:**
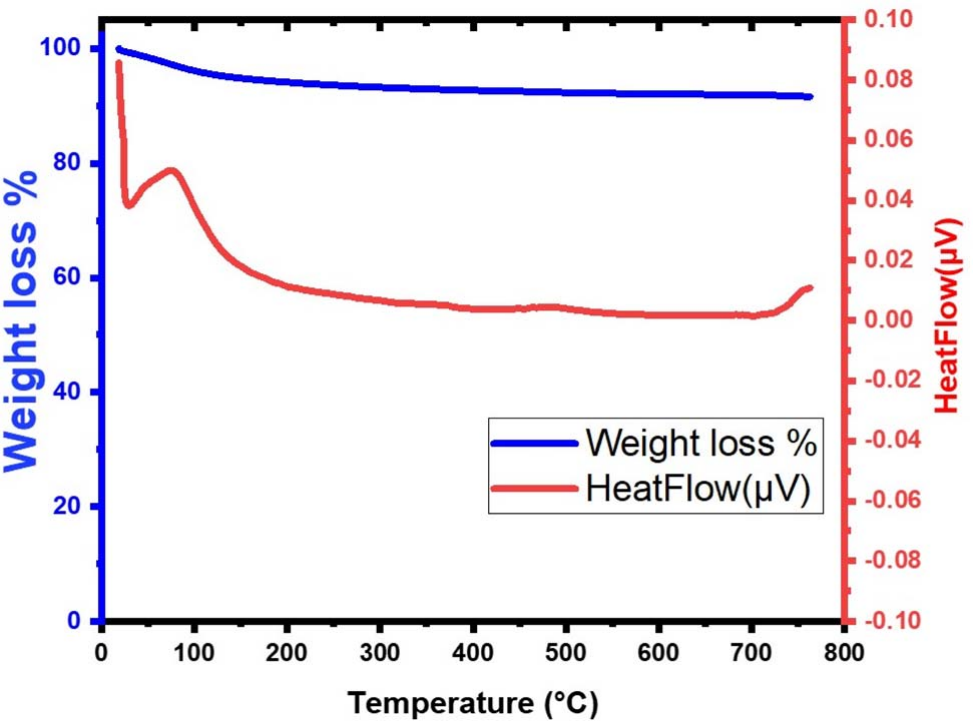
Thermal behavior of OCP.

The thermogravimetric (weight loss) curve (Fig. 5) exhibits an initial reduction in mass at comparatively low temperatures, a phenomenon that is characteristic of materials that possess either structural or adsorbed moisture. In the case of octacalcium phosphate pentahydrate, this behavior aligns with the liberation of hydration water from its crystalline architecture. The gradual incline of the curve signifies that the dehydration process transpires in stages rather than as a singular, abrupt occurrence, implying that water molecules are associated with varying degrees of binding strength within the structural framework. The differential scanning calorimetry (DSC) curve (Fig. 4) reveals an endothermic response at low to moderate temperatures, indicative of the energy required for the extraction of water. This endothermic phenomenon reinforces the notion that dehydration constitutes the predominant process. After this region, the heat flow reaches a plateau, signifying that no significant phase transformation transpires beyond the expulsion of water.

In summary, the amalgamated data regarding weight loss and heat flow substantiate that octacalcium phosphate pentahydrate experiences dehydration when subjected to thermal treatment, resulting in the elimination of structural water while the inorganic matrix maintains a relatively stable condition within the examined temperature spectrum. The progressive nature of the mass reduction indicates that water is released in a gradual manner, which is emblematic of hydrated calcium phosphate phases.

### 3.4 Morphological analysis (SEM)

**Fig. 6:**
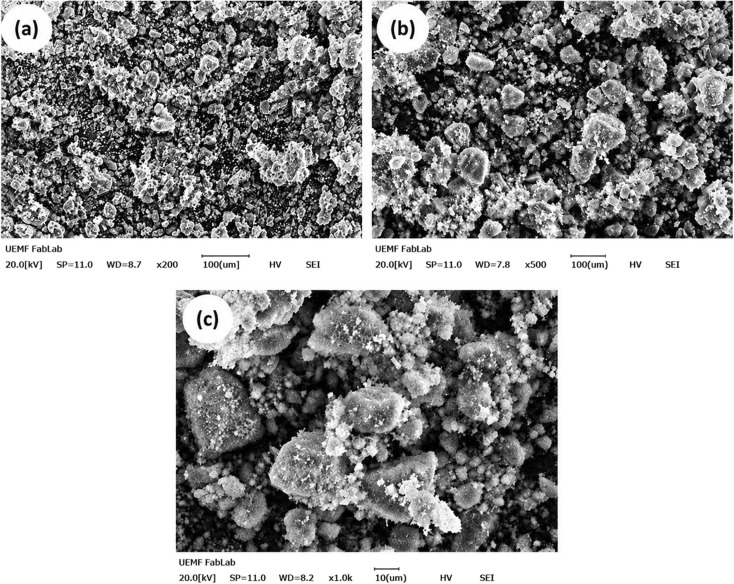
SEM images of the synthesized material: (a) porous morphology (×200), (b) plate-like crystal aggregates (×500), and (c) rough nanoscale surface (×1000).

In the analysis utilizing Scanning Electron Microscopy (SEM), the octacalcium phosphate material demonstrates a well-defined and homogeneous morphology characterized by a porous and textured surface, which increases the available active sites and surface accessibility. This structural feature facilitates the diffusion of methylene blue molecules toward the adsorption sites, thereby enhancing the overall adsorption efficiency, and also exhibits a hierarchical architecture that is advantageous for antimicrobial activity. At a low magnification level of 200 (Fig. 6(a)), the material presents a homogeneous, extensively porous framework, which facilitates a substantial surface area for bacterial engagement. Upon increasing the magnification to 500 (Fig. 6(b)), this framework is shown to be comprised of aggregates of slender, plate-like crystals characterized by sharp geometric edges, a morphology recognized for its ability to induce physical disruption of bacterial cell membranes. Ultimately, the nanoscale surface topology observed at a magnification of 1000 (Fig. 6(c)), featuring attributes suggestive of active dissolution, substantiates the presence of a highly reactive surface capable of altering the local ionic milieu and optimizing contact with microbial cells. This 3-dimensional morphology, macro-porous, micro-platelet, and nano-textured, contributes synergistically to the potential antimicrobial efficacy of OCP through an interplay of physical and physicochemical mechanisms.^31–33^

### 3.5 Microstructural analysis (BET)

**Fig. 7:**
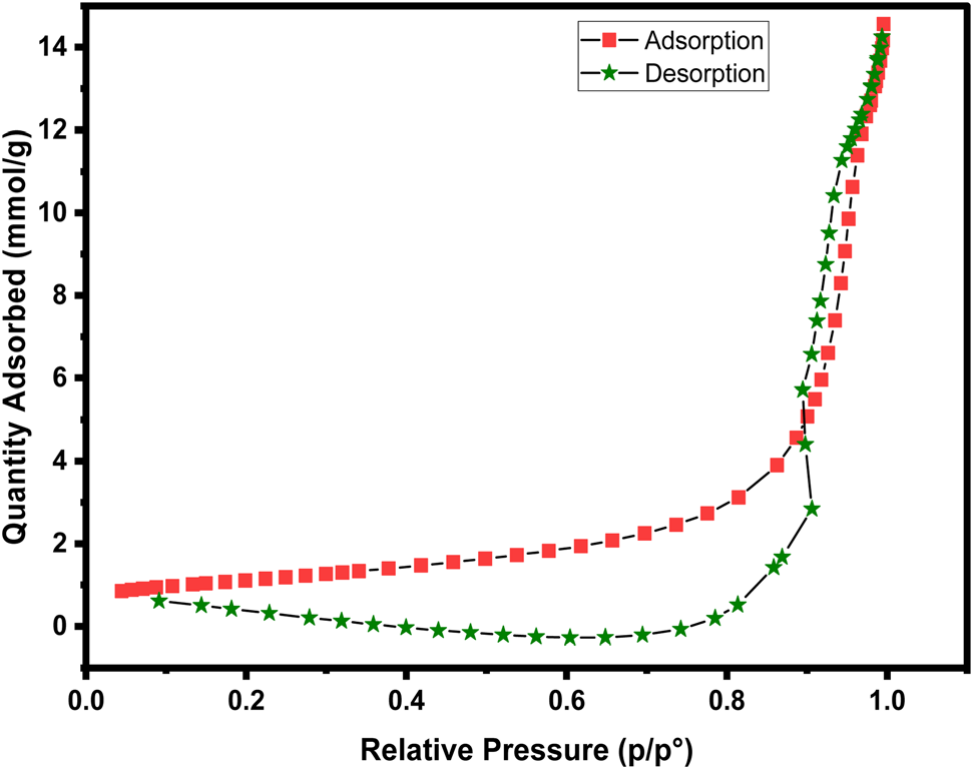
*t*-Plot analysis of octacalcium phosphate pentahydrate.

The N_2_ adsorption–desorption isotherm of the synthesized OCP is shown in Fig. 7. According to the IUPAC classification, the isotherm can be assigned to Type IV, which is characteristic of mesoporous materials. The presence of a hysteresis loop at high relative pressures *P*/*P*_0_ > 0.8 indicates capillary condensation within mesopores. The shape of the hysteresis loop resembles the H3 type, generally associated with slit-shaped pores formed by aggregates of plate-like particles.^34,35^ This observation is consistent with the layered crystal structure and platelet-like morphology of OCP observed by SEM. The mesoporous nature of the material is expected to facilitate mass transfer and provide accessible adsorption sites, contributing to its adsorption performance toward methylene blue. Fig. 7 shows the *t*-plot analysis of octacalcium phosphate (OCP), which reveals a total surface area (BET) of 87.80 m^2^ g^−1^, accompanied by an external surface area of 85.66 m^2^ g^−1^. The micropore area and micropore volume are exceedingly minimal, recorded at 2.13 m^2^ g^−1^ and 0.00126 cm^3^ g^−1^, respectively, suggesting that the material is fundamentally non-microporous. This predominantly external surface characteristic is beneficial for antimicrobial applications,^36^ as it offers numerous contact sites for interaction with microbial cells, thus augmenting the inhibitory efficacy of the material;^36–38^ and also is advantageous for the removal of dyes from aqueous solutions.

### 3.6 Hirshfeld analysis

**Fig. 8:**
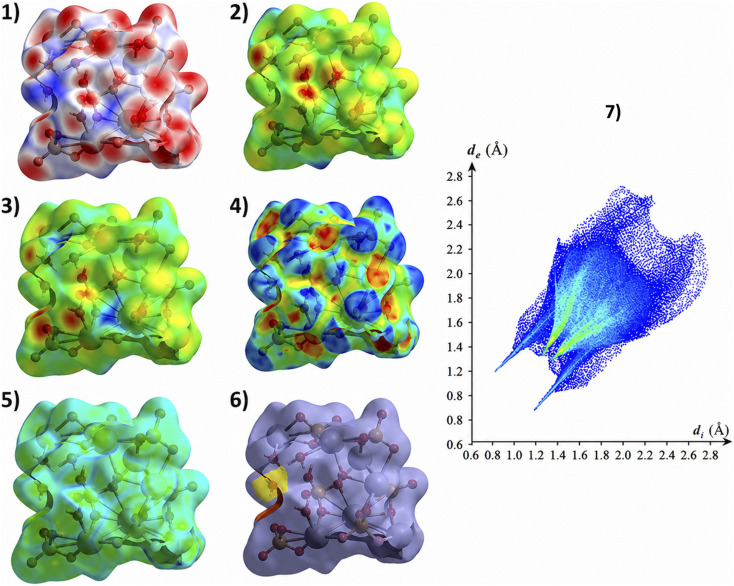
Hirshfeld surface analysis of OCP.

The analysis of the Hirshfeld surface for OCP pentahydrate (Fig. 8) elucidates that the arrangement of the crystal lattice is chiefly influenced by hydrogen bonding interactions. The *d*_norm_ surface (1) reveals multiple pronounced red regions, signifying close contacts that are shorter than the cumulative van der Waals radii, which are primarily ascribed to robust O–H⋯O hydrogen bonds that involve both the host molecule and the lattice water molecules. The *d*_i_ (2) and *d*_e_ (3) mappings further corroborate the bifunctional role of oxygen atoms as both hydrogen-bond donors and acceptors, thereby reinforcing the establishment of an extensive three-dimensional hydrogen-bonded network. The shape index (5) and curvedness (6) surfaces do not exhibit notable complementary flat regions, indicating an absence of π–π stacking interactions and emphasizing the preeminence of localized, directional contacts. The two-dimensional fingerprint plot (7) illustrates sharp peaks at diminished *d*_i_ and *d*_e_ values, which are indicative of strong O⋯H/H⋯O interactions, whereas broader regions correspond to the weaker H⋯H contacts that contribute to the overall stabilization of the packing. The computed globularity (0.702) and low asphericity (0.038) signify a relatively compact and nearly isotropic molecular configuration within the lattice. Collectively, the results derived from the Hirshfeld surface analysis indicate that the structural integrity of OCP pentahydrate is predominantly governed by extensive hydrogen bonding, with van der Waals interactions assuming a secondary significance. Beyond its structural description, Hirshfeld surface analysis provides a quantitative assessment of intermolecular interaction contributions within the crystal packing, emphasizing the dominant role of directional hydrogen bonding in stabilizing the OCP lattice. This interaction landscape is directly relevant to the functional behavior of the material, as the hydrogen-bonded network modulates surface polarity, hydration dynamics, and the availability of reactive oxygen sites. Such features are critical in governing adsorption processes in aqueous environments by enhancing dye–surface interactions through electrostatic attraction, hydrogen-bonding affinity, and interfacial charge localization. In addition, the same surface characteristics may contribute to the antimicrobial performance of OCP, where strong interfacial hydrogen bonding and polar surface domains can promote adhesion to microbial cell walls, disrupt membrane integrity through localized interfacial stress, and interfere with fungal and bacterial surface hydration layers. Therefore, Hirshfeld surface analysis provides mechanistic insight linking crystal packing features to adsorption and antimicrobial functionality.

### 3.7 Adsorption efficacy

#### 3.7.1 Effect of contact duration and effect of pH

The investigation of methylene blue adsorption on Ca_8_(HPO_4_)_2_(PO_4_)_4_·5H_2_O in solution necessitates the evaluation of the contact time that corresponds to either an equilibrium state of substrate saturation of the support or an equilibrium of adsorption/desorption. Adsorption tests were conducted on solutions of MB with an initial concentration of 10 mg L^−1^ at a temperature of 25 °C for a duration ranging from 15 to 165 minutes to assess the impact of contact time on the adsorption of the MB on the selected adsorbent. Adsorption isotherms for the adsorbent might be established by figuring out the contact time, which corresponds to the adsorption equilibrium. Understanding this duration is crucial for determining the maximal adsorption capacity and the kind of adsorption that can take place in a mono- or multi-layer system.

**Fig. 9:**
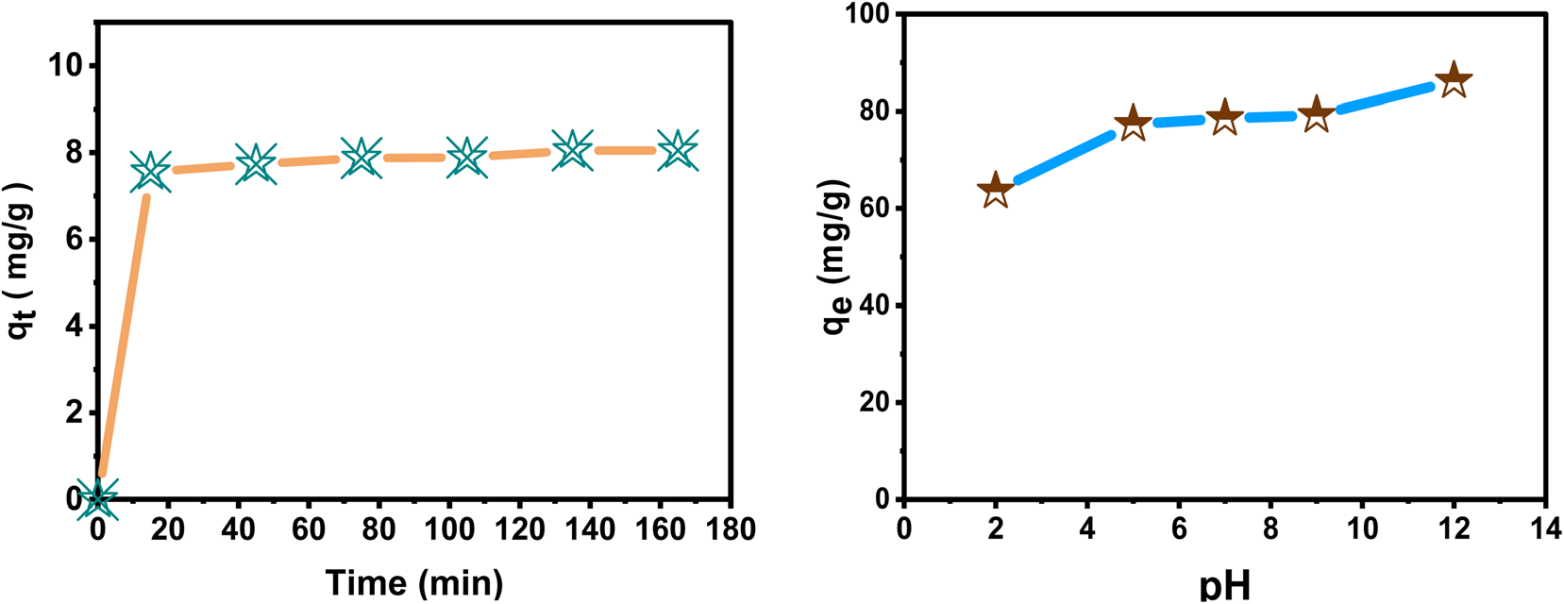
Effect of MB contact time (*C*_i_ = 10 mg L^−1^, *m* = 0.01 g, *T* = 25 °C) and effect of pH (*C*_i_ = 100 mg L^−1^, *m* = 0.1 g, *T* = 25 °C) on Ca_8_(HPO_4_)_2_(PO_4_)_4_·5H_2_O.

The findings in Fig. 9 (left) demonstrate that the adsorbed amount of MB rises quickly in the first 15 minutes to 7.87 mg g^−1^ and then stabilizes at a level close to equilibrium after 80 minutes. This demonstrates how quickly the dye is adsorbed by the utilized adsorbent. The adsorption stays steady after that. This is because there are a lot of empty adsorption sites on the phosphate surface during the first stage of adsorption. The development of repulsive interactions between the MB molecules in the aqueous phase (free) and those on the solid surface (adsorbed) makes it challenging to fill the remaining empty exterior sites over time. Furthermore, the mass transfer between the liquid and solid phases would gradually decrease due to the medium size of the MB molecules and their easy diffusion into the interior pores until they are saturated. As a result, the adsorption rate drops, and after 75 minutes, a plateau that represents the steady state is shown.

One of the most important elements in the adsorption process is pH.^39,40^ It has been studied how the pH of the MB affects the adsorption behavior in the pH range of 2–12. The adsorption behavior of MB with pH variation makes it evident that basic pH has a larger adsorption capacity than acidic pH (Fig. 9 (right)). MB is a dye that is basic. Reduced ions (CH^+^) and cations (C^+^) are produced by MB in water. The sorption of basic MB dyes is favored when the pH of the solution is higher than the zero charge point of Ca_8_(HPO_4_)_2_(PO_4_)_4_·5H_2_O because this raises the negative charge density on the compound surface. Additionally, under an acidic pH, MB will get protonated, resulting in a positive charge density.^41^

#### 3.7.2 Effect of initial dye concentration and adsorption isotherm

**Fig. 10:**
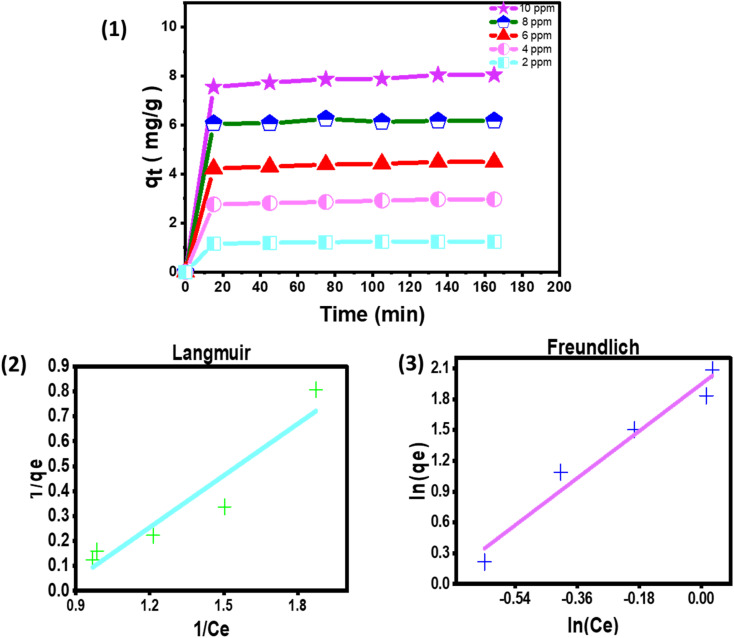
(1) Effect of initial MB concentration on the adsorption capacity of OCP (*V* = 10 mL, *m* = 0.01 g, *T* = 25 °C), and comparison of MB adsorption isothermal models on OCP; (2) Langmuir and (3) Freundlich.

The initial concentration of the adsorbate serves as a critical driving force to surmount any limitations associated with mass transfer between the two distinct phases, namely the liquid and the solid.^42,43^Fig. 10(1) elucidates the influence of the initial concentration of methylene blue (MB) on its adsorption by Ca_8_(HPO_4_)_2_(PO_4_)_4_·5H_2_O. From the analysis of Fig. 10, it is evident that as the initial concentration of the dye escalates from 2 to 10 ppm, the removal efficiency of MB correspondingly increases from 70% to 89%. This enhancement in MB adsorption capacity can likely be attributed to the augmentation of the mass gradient between the adsorbate solution and Ca_8_(HPO_4_)_2_(PO_4_)_4_·5H_2_O as the initial dye concentration rises. The isothermal models of Langmuir and Freundlich have been employed to rigorously investigate the dynamics of adsorption. These models facilitate the examination of the relationship between the adsorption capacity of MB and equilibrium concentration. The respective linear equations corresponding to the isotherm models are delineated in Table 4. The linear regression analysis of the Langmuir and the Freundlich isotherms is depicted in Fig. 10(2) and (3). The adsorption of MB onto Ca_8_(HPO_4_)_2_(PO_4_)_4_·5H_2_O was more accurately described by the Freundlich isotherm, yielding a correlation coefficient (*R*^2^) of 0.9896, which surpasses the values obtained with the Langmuir model. The Freundlich sorption isotherm is posited to occur on a heterogeneous surface through a multilayer sorption mechanism.^44^

**Table 4:** Isotherm parameters and related correlation coefficients for the MB dye's adsorption by Ca_8_(HPO_4_)_2_(PO_4_)_4_·5H_2_O

Model	Parameters	Value
Langmuir	*q*_m_ (mg g^−1^)	1.9378
	*K*_L_ (L mg^−1^)	0.9453
	*R* ^2^	0.91
Freundlich	*K*_F_ (mg^1−(1/*n*)^ L^1/*n*^ g^−1^)	7.1189
	*n*	2.478
	*R* ^2^	0.9896

#### 3.7.3 Adsorption thermodynamics

**Fig. 11:**
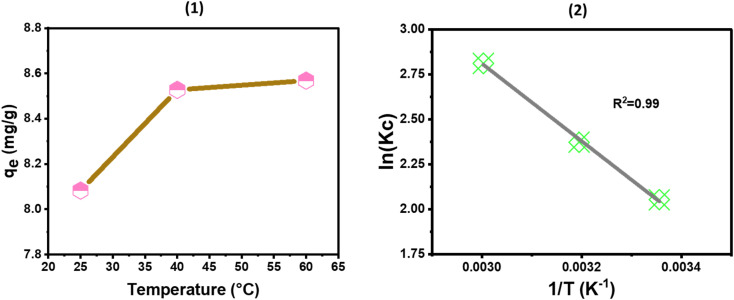
(1) Influence of temperature on the adsorption and (2) the plot of ln(*K*_c_) *vs.* (1/*T*) for MB dye by Ca_8_(HPO_4_)_2_(PO_4_)_4_·5H_2_O.

Fig. 11 demonstrates how temperature affects the equilibrium adsorption capacity of MB onto Ca_8_(HPO_4_)_2_(PO_4_)_4_·5H_2_O. The plots indicate an increase in the equilibrium adsorption capacity concurrent with an elevation in temperature from 25 °C to 60 °C. The thermodynamic parameters calculated for methylene blue adsorption onto OCP are summarized in Table 5. The positive enthalpy change Δ*H*° = 18.25 kJ mol^−1^ indicates that the adsorption process is endothermic in nature, suggesting that increasing temperature favors dye uptake. The positive entropy change Δ*S*° = 75.94 J mol^−1^ K^−1^ reflects an increase in randomness at the solid–liquid interface during adsorption. Furthermore, the negative Gibbs free energy values obtained at all investigated temperatures, Δ*G*° = −5.75, −6.37, and −7.67 kJ mol^−1^ at 298, 313, and 333 K, respectively, confirm the spontaneous nature of the adsorption process. The increasingly negative Δ*G*° values with rising temperature further indicate that adsorption becomes more thermodynamically favorable at higher temperatures.^45^

**Table 5:** The thermodynamic parameters of MB dye adsorption onto Ca_8_(HPO_4_)_2_(PO_4_)_4_·5H_2_O

Adsorbent	Adsorbate	Δ*H*° (kJ mol^−1^)	Δ*S*° (J mol^−1^ K^−1^)	Δ*G*° (kJ mol^−1^)		
				298 K	313 K	333 K
OCP	MB	18.2498	75.9372	−5.750	−6.369	−7.67

#### 3.7.4 Adsorption kinetics

**Fig. 12:**
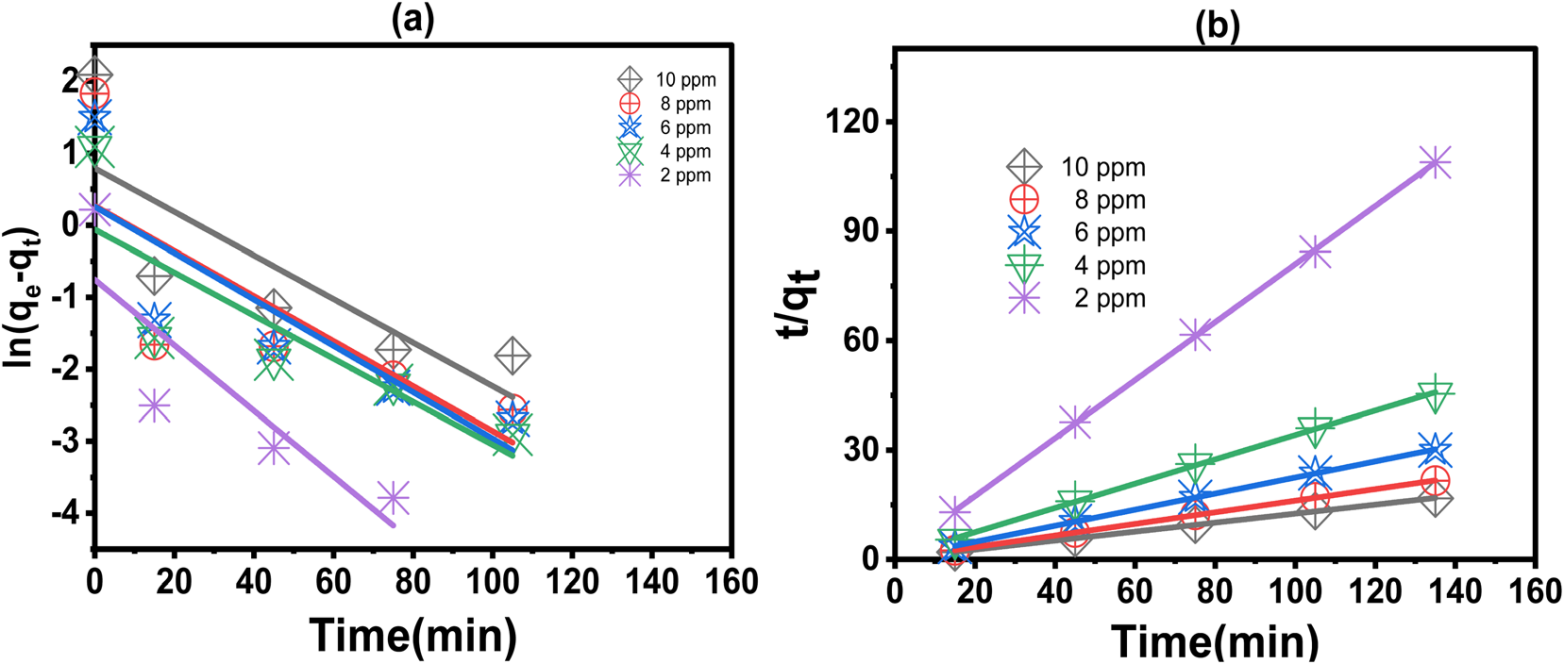
Kinetic models (a) pseudo-first-order and (b) pseudo-second-order for the adsorption of MB dye with different initial concentrations (2–10 mg L^−1^) using 0.01 g of the synthesized compound.

To scrutinize the sorption kinetics of MB on Ca_8_(HPO_4_)_2_(PO_4_)_4_·5H_2_O, both the pseudo-first-order model and the pseudo-second-order model were utilized for various initial MB concentrations.^46,47^ The linear fitting of the pseudo-first-order (PFO) model and pseudo-second-order (PSO) kinetics is illustrated in Fig. 12. The constants for these models are presented in Table 6. A comparison of the correlation coefficients (*R*^2^) for the pseudo-first-order and pseudo-second-order kinetic models reveals that the experimental data align more closely with the pseudo-second-order model, yielding a superior correlation coefficient (*R*^2^ > 0.99). The second-order kinetic sorption process posits that the sorption mechanism is influenced by the chemical sorption characteristic of the surface adsorption mechanism, whereas the first-order kinetic sorption model presumes that the adsorption process is predominantly governed by the diffusion step. In the context of surface sorption, the interaction of electron sharing or transfer between the adsorbate and the adsorbent facilitates chemical sorption.^48^ These observations suggest that the predominant mechanism of MB sorption is primarily governed by chemical sorption.^49^

**Table 6:** Pseudo-first-order and pseudo-second-order kinetic parameters for MB adsorption on OCP

Dye concentration (ppm)	*q*_e_ (exp) (mg g^−1^)	PFO			PSO		
		*q*_e_ (cal)	*K*_1_	*R* ^2^	*q*_e_ (cal)	*K*_2_	*R* ^2^
10	8.1177	0.80223	0.02930	0.6700	8.1058	0.0702	0.99969
8	6.25340	0.25740	0.03030	0.598	6.247	0.14	0.99982
6	4.49137	0.2031	0.03334	0.7103	4.510	0.130	0.99977
4	2.97002	−0.04901	0.0374	0.7301	2.9888	0.141	0.9995
2	1.23978	−0.7130	0.04567	0.7503	1.244	0.45	0.99983

### 3.8 Adsorption mechanism

**Fig. 13:**
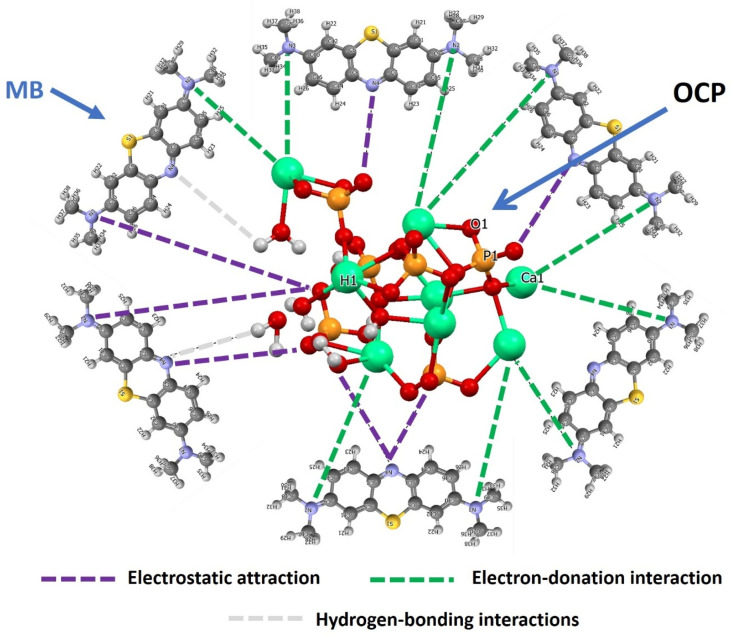
Proposed interaction mechanism between OCP and methylene blue dye.

The proposed interaction mechanism is illustrated in Fig. 13. Methylene Blue (MB) interacts with well-defined atomic sites of the adsorbent and the dye molecule, octacalcium phosphate pentahydrate (Ca_8_H_2_(PO_4_)_6_·5H_2_O). Oxygen atoms from phosphate (PO_4_^3−^) and hydrogen phosphate (HPO_4_^2−^) groups, where oxygen carries partial negative charges, are the dominant adsorption sites on the OCP surface. The oxygen atoms interact electrostatically with the positively charged quaternary nitrogen (N^+^) present in the central aromatic ring of methylene blue.

Moreover, exposed Ca^2+^ ions on OCP may take part in coordination interactions with electron-donating nitrogen atoms from the dimethylamino fragments (–N(CH_3_)_2_) localizing in the dye structure. Hydrogen bond interactions may also play a role since hydrogen atoms from structural water molecules or surface hydroxyl groups of OCP can interact with nitrogen atoms of MB. The most significant charge-transfer filling is due to electron transfer from phosphate O atoms within the anion activating methylene blue (MB), with another contribution coming from Ca^2+^–nitrogen coordination and hydrogen bonding.

### 3.9 Comparative study

Table 7 compares the adsorption performance of the synthesized OCP with several mineral-based adsorbents reported in the literature. Although the adsorption capacity of OCP (7.87 mg g^−1^) is lower than that reported for kaolinite and biogenic calcium carbonate, direct comparison should be made with caution due to significant differences in the experimental conditions. In the present study, adsorption experiments were conducted using a low initial methylene blue concentration of 10 ppm and a very small adsorbent mass of 0.01 g, whereas higher adsorption capacities reported in the literature were often obtained at higher dye concentrations and/or with larger adsorbent dosages. Under these conditions, the synthesized OCP exhibited an adsorption capacity comparable to those reported for non-calcined phosphate 9.54 mg g^−1^ and hydroxyapatite 4 mg g^−1^, which are chemically related phosphate-based materials. OCP combines adsorption capability with antibacterial activity and bioactivity, providing multifunctional properties that are generally not considered in conventional adsorbent materials. These results demonstrate that phase-pure OCP represents a promising multifunctional material for both environmental and biomedical applications.^50,51^

**Table 7:** Adsorption capacities of MB onto various adsorbents

Adsorbents	Conditions	Adsorption capacity *q*_e_ (mg g^−1^)
Non-calcined phosphate^50^	pH 9; *C*_i_ 10 mg L^−1^, dose 1 g L^−1^; *t* 180 min	9.54
Hydroxyapatite^51^	*m* 0.2 g; pH 9; *C*_i_ 20 mg L^−1^	4
This study	*m* 0.01 g; *C*_i_ 10 mg L^−1^; *T* 25 °C; pH 6.5; *t* 165 min	7.87

### 3.10 Antibacterial efficacy

In this part, the antibacterial activity of Ca_8_(HPO_4_)_2_(PO_4_)_4_·5H_2_O, was evaluated. Table 8 shows the acquired results.

Antibacterial activity of OCP in mg mL^−1^

**Table:** 

		*S. aureus*	*B. cereus*	*L. monocytogenes*	*M. flavus*
OCP	MIC	0.14 ± 0.03	0.14 ± 0.04	0.28 ± 0.06	0.28 ± 0.06
	MBC	0.29 ± 0.04	0.29 ± 0.02	0.56 ± 0.02	0.56 ± 0.07
Streptomycin	MIC	0.04 ± 0.005	0.09 ± 0.008	0.17 ± 0.03	0.17 ± 0.04
	MBC	0.09 ± 0.003	0.17 ± 0.009	0.34 ± 0.04	0.34 ± 0.04
Ampicillin	MIC	0.25 ± 0.03	0.25 ± 0.06	0.25 ± 0.07	0.37 ± 0.06
	MBC	0.37 ± 0.04	0.37 ± 0.08	0.37 ± 0.01	0.49 ± 0.03

**Table:** 

		*P. aeruginosa*	*E. coli*	*S. typhimurium*	*E. cloacae*
OCP	MIC	0.14 ± 0.01	0.28 ± 0.03	0.27 ± 0.03	0.28 ± 0.03
	MBC	0.29 ± 0.04	0.56 ± 0.07	0.56 ± 0.06	0.56 ± 0.06
Streptomycin	MIC	0.34 ± 0.04	0.26 ± 0.03	0.17 ± 0.01	0.17 ± 0.01
	MBC	0.68 ± 0.09	0.52 ± 0.08	0.34 ± 0.07	0.34 ± 0.07
Ampicillin	MIC	0.74 ± 0.09	0.37 ± 0.09	0.37 ± 0.09	0.25 ± 0.04
	MBC	1.24 ± 0.1	0.74 ± 0.08	0.49 ± 0.04	0.49 ± 0.04

The OCP demonstrated significant inhibitory activity against all examined bacterial strains, with minimum inhibitory concentration (MIC) values ranging from 0.14 to 0.28 mg mL^−1^ for the majority of strains. It exhibited comparatively enhanced effects against Gram-positive bacteria, evidenced by lower MIC and minimum bactericidal concentration (MBC) values in contrast to various Gram-negative strains, where bactericidal concentrations were typically elevated (up to 0.56 mg mL^−1^). Streptomycin revealed superior potency overall, manifesting lower MIC and MBC values across almost all tested organisms. Ampicillin displayed inconsistent efficacy and was less effective against certain strains, most notably *P. aeruginosa*, which necessitated the highest concentrations. Notably, OCP exhibited MIC values of 0.14 mg mL^−1^ against *S. aureus*, *B. cereus*, and *P. aeruginosa*, which were comparable to or lower than those obtained for ampicillin against the same strains. Nevertheless, streptomycin generally showed greater antibacterial potency, as reflected by its lower MIC and MBC values for most bacterial species. These findings indicate that OCP possesses promising antibacterial activity, although it does not surpass the efficacy of the most active conventional antibiotic tested. Although the exact antimicrobial mechanism of OCP was not investigated in the present study, its activity may arise from several factors reported for calcium phosphate-based materials, including the release of calcium and phosphate ions, surface-mediated interactions with microbial cell membranes, and local physicochemical changes at the material–microorganism interface. These effects may alter membrane permeability and cellular homeostasis, thereby inhibiting microbial growth. Compared with conventional calcium phosphate biomaterials such as hydroxyapatite (HA) and β-tricalcium phosphate (β-TCP), OCP may offer advantages due to its higher solubility and ion-release capability, which can enhance interactions with microbial cells. Previous studies have shown that significant antimicrobial activity in calcium phosphates is often achieved through ion substitution (Ag, Cu, or Zn) or incorporation of antibacterial agents, whereas undoped calcium phosphates generally exhibit weaker antimicrobial effects. Therefore, the antibacterial and antifungal activities observed for OCP in the present study highlight its potential as a bioactive calcium phosphate material with intrinsic antimicrobial properties.^52–56^

### 3.11 Antifungal effect

OCP exhibited antifungal efficacy against all examined species (Table 9), with minimum inhibitory concentration (MIC) values ranging from 0.18 to 0.78 mg mL^−1^. It demonstrated superior effectiveness against *A. fumigatus* (MIC = 0.19 ± 0.03 mg mL^−1^), *A. versicolor* (MIC = 0.18 ± 0.03 mg mL^−1^), *P. ochrochloron* (MIC = 0.18 ± 0.04 mg mL^−1^), and *P. funiculosum* (MIC = 0.19 ± 0.03 mg mL^−1^), whereas elevated concentrations were necessary to inhibit *A. niger*, *P. verrucosum*, and *T. viride*. The respective minimum fungicidal concentration (MFC) values were generally approximately double the MICs, suggesting a concentration-dependent fungicidal mechanism of action.

Antifungal activity of OCP in mg mL^−1^

**Table:** 

		*A. fumigatus*	*A. versicolor*	*A. ochraceus*	*A. niger*
OCP	MIC	0.19 ± 0.03	0.18 ± 0.03	0.39 ± 0.06	0.78 ± 0.07
	MFC	0.39 ± 0.06	0.39 ± 0.04	0.79 ± 0.04	1.56 ± 0.15
Ketoconazole	MIC	0.21 ± 0.04	0.21 ± 0.03	0.16 ± 0.02	0.21 ± 0.04
	MFC	0.51 ± 0.03	0.53 ± 0.04	0.21 ± 0.01	0.51 ± 0.06
Bifonazole	MIC	0.15 ± 0.04	0.11 ± 0.01	0.14 ± 0.01	0.16 ± 0.03
	MFC	0.21 ± 0.03	0.21 ± 0.04	0.21 ± 0.03	0.21 ± 0.04

**Table:** 

		*P. ochrochloron*	*P. funiculosum*	*P. verrucosum*	*T. viride*
OCP	MIC	0.18 ± 0.04	0.19 ± 0.03	0.40 ± 0.05	0.40 ± 0.06
	MFC	0.40 ± 0.05	0.40 ± 0.05	0.79 ± 0.03	0.79 ± 0.07
Ketoconazole	MIC	1.01 ± 0.09	0.21 ± 0.07	0.21 ± 0.04	1.00 ± 0.1
	MFC	1.50 ± 0.12	0.51 ± 0.05	0.30 ± 0.03	1.50 ± 0.1
Bifonazole	MIC	0.21 ± 0.04	0.21 ± 0.04	0.11 ± 0.01	0.14 ± 0.01
	MFC	0.24 ± 0.03	0.24 ± 0.01	0.21 ± 0.01	0.21 ± 0.03

In comparison to the reference antifungal agents, bifonazole exhibited the most pronounced and consistent antifungal activity, manifesting the lowest MIC and MFC values across the majority of strains assessed. Ketoconazole revealed inconsistent efficacy, demonstrating substantial activity against certain species but significantly diminished effects against *P. ochrochloron* and *T. viride*. Collectively, OCP manifested moderate, broad-spectrum antifungal potential, albeit inferior in potency compared to bifonazole under the conditions evaluated.

### 3.12 Evolution of ionic concentrations during *in vitro* immersion

**Fig. 14:**
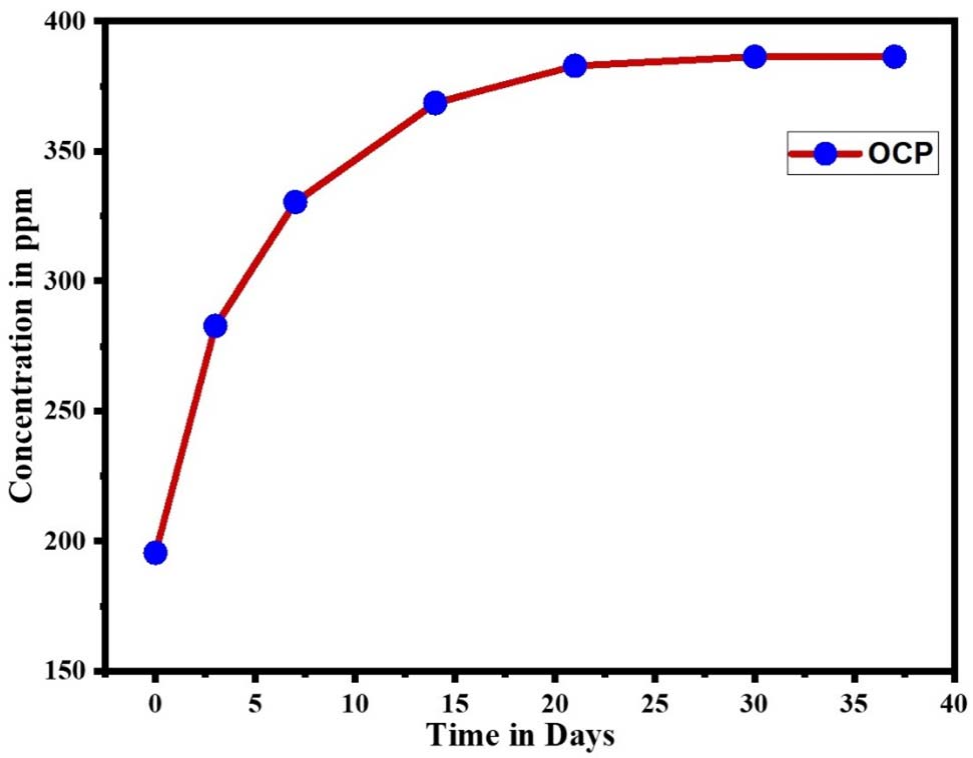
Evolution of ionic concentration in SBF as a function of immersion time for the OCP material.

The evolution of ionic concentration in SBF in the presence of OCP (Fig. 14) reveals a progressive ion-exchange process between the material and the surrounding medium.^57^ The marked increase in ionic concentration at early immersion times can be attributed to the partial dissolution of the OCP surface, resulting in the release of calcium- and phosphate-containing ionic species into the solution.^58^ As immersion time increases, the rate of concentration increase gradually decreases, suggesting a reduction in dissolution kinetics and the progressive establishment of equilibrium between the material surface and the surrounding medium. The stabilization of ionic concentration at longer immersion times indicates that ion exchange becomes limited with time. While this behavior may be associated with surface modifications occurring during immersion, the present data do not provide direct evidence of apatite formation, and additional surface characterization would be required to confirm such a process. Nevertheless, the observed controlled ion-release behavior is of particular interest for biomedical applications, as the gradual release of calcium and phosphate ions may contribute to the creation of a favorable local environment for bone tissue regeneration. Such characteristics make OCP a promising candidate for applications including bone graft substitutes, bone defect fillers, bioactive coatings for metallic implants, and tissue engineering scaffolds, where controlled ionic exchange can support biological responses and tissue integration.^59^

## 4 Conclusion

In this work, we have reported the synthesis of the octacalcium phosphate pentahydrate, and characterized it using XRD analysis combined with Rietveld refinement, which demonstrates the phase purity of the synthesized product. Also, the FTIR analysis was performed to identify the presence of the main phosphate groups (HPO_4_), and water molecules. The TGA indicates that the thermal decomposition of the compound leads to the release of water molecules. The Scanning Electron Microscopy reveals that the morphology of the synthesized product consists of uniformly distributed spherical particles. The nitrogen adsorption–desorption isotherms, analyzed using the BET model, revealed a specific surface area of 87.80 m^2^ g^−1^, confirming its porous nature. The synthesized product was evaluated for its ability to remove organic dye (MB) by adsorption, and it performed well under a variety of conditions, particularly high temperatures and basic medium with modest amounts of the synthesized product. Furthermore, the product exhibited a good antibacterial and antifungal activity, underlining its bioactivity potential and functional performance, and demonstrating its ability to regulate ionic exchange in physiological environments. While these *in vitro* results are promising, further studies are required to evaluate the material's biocompatibility with human cells and its performance in dynamic flow-through systems for water treatment.

## Author contributions

Mohammed Zerrouk: writing – original draft, writing – review and editing, methodology, formal analysis, investigation, conceptualization. Imane Haydari: software, investigation, methodology, validation. Mohammed Mesrar: data curation, software, investigation. Mohammed Lachkar: supervision. Lamy Mamdoh Mohamed Hamed, Ahmad El-Harairy, and Khalil Azzaoui: writing – review and editing, validation, project administration, resources. Belkheir Hammouti: formal analysis. Rachid Ouarsal: supervision, validation.

## Conflicts of interest

There are no conflicts to declare.

## Data Availability

Comprehensive and explicit characterizations of the utilized materials and instruments are articulated within the materials and methods segment of the manuscript. Furthermore, the acquired data is substantiated through the citation of the figures and tables present in the manuscript. Overall, all data that were produced or examined throughout this investigation are encompassed within this published article.
