# Correction: A multifunctional octacalcium phosphate pentahydrate with dual environmental and biomedical functions: efficient dye removal, potent antimicrobial activity, and ionic regulation in physiological media

**DOI:** 10.1039/d6ra90102f

**Published:** 2026-08-18

**Authors:** Mohammed Zerrouk, Imane Haydari, Mohammed Mesrar, Belkheir Hammouti, Mohammed Lachkar, Lamy Mamdoh Mohamed Hamed, Ahmed El-Harairy, Khalil Azzaoui, Rachid Ouarsal

**Affiliations:** a Engineering Laboratory of Organometallic, Molecular Materials, and Environment, Faculty of Sciences, University Sidi Mohamed Ben Abdellah Fez 30000 Morocco Mohammed.zerrouk@usmba.ac.ma; b Materials Science, Energy and Nanoengineering (MSN) Department, Mohammed VI Polytechnic University Ben Guerir Morocco; c Signals, Systems and Components Laboratory (LSSC), Faculty of Sciences and Technologies of Fez, Sidi Mohamed Ben Abdellah University B. P. 2022 Fez Morocco; d Euro-Mediterranean University of Fes BP 15 Fes 30070 Morocco; e Department of Environment and Agricultural Natural Resources, College of Agricultural and Food Sciences, King Faisal University Al-Ahsa Saudi Arabia lamy.hamed@kfu.edu.sa; f Department of Chemical and Biomolecular Engineering, College of Engineering, University of Nebraska-Lincoln Lincoln NE 68588 USA ael-harairy2@nebraska.edu; g Department of Soils and Water, Faculty of Agriculture, Damietta University New Damietta Damietta 34517 Egypt; h Laboratory of Industrial Engineering, Energy and the Environment (LI3E), SUPMTI Rabat Morocco

## Abstract

Correction for ‘A multifunctional octacalcium phosphate pentahydrate with dual environmental and biomedical functions: efficient dye removal, potent antimicrobial activity, and ionic regulation in physiological media’ by Mohammed Zerrouk *et al.*, *RSC Adv.*, 2026, **16**, 32849–32864, https://doi.org/10.1039/d6ra01926a.

The authors regret that, in the published article and author contributions, an author name was incorrectly given as Ahmad El-Harairy. In both cases, the correct spelling should be Ahmed El-Harairy, as shown in this notice.

The Royal Society of Chemistry apologises for these errors and any consequent inconvenience to authors and readers.

